# A Growing Lesion of the Lip

**Published:** 2015-04-18

**Authors:** Andrew A. Marano, Paul J. Therattil, Stephen L. Viviano, Ramazi O. Datiashvili

**Affiliations:** Division of Plastic and Reconstructive Surgery, Department of Surgery, Rutgers New Jersey Medical School, Newark, New Jersey

**Keywords:** lip lesion, lip mass, mucocele, salivary gland swelling, chronic lip trauma

**Figure:**
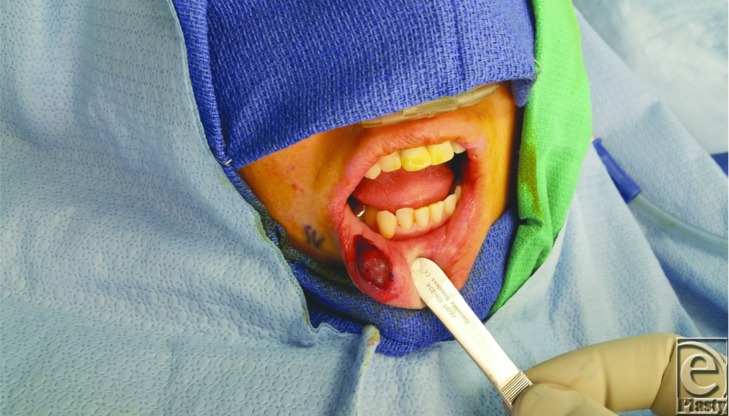


## DESCRIPTION

A 29-year-old woman with bipolar disorder presented to the plastic surgery clinic with a gradually growing mass of the lower lip. The patient reported that the lesion was irritating and she frequently found herself biting it. It had waxed and waned in size over several years.

## QUESTIONS

1. **What is the differential diagnosis for a growing lip mass?**
2. **What is the pathogenesis and classification of oral mucoceles?**
3. **What are the management options?**
4. **What is the risk of recurrence after removal?**

## DISCUSSION

There is a broad differential diagnosis for a growing mass of the lower lip. The 2 most common lesions are traumatic fibromas, or focal fibrous hyperplasia, and mucoceles.^1^ Both lesions are smooth, spherical, and irritating and have the color of the oral mucosa. Furthermore, both can be caused by trauma from biting and thus appear along the line of occlusion along the lower lip. Oral mucoceles tend to be soft and fluctuant whereas fibromas are firm and nodular, but definitive diagnosis requires excision and histological analysis. Other less common lesions with similar presentation include lipomas, mucus retention cysts, sialoliths, phleboliths, and salivary gland neoplasms.^2^ The typical course for an oral mucocele is spontaneous rupture from trauma shortly after formation, followed by release of viscous fluid.^3^ The resultant decrease in size often gives patients the perception that healing has occurred, but fluid reaccumulates and the lesion recurs. This explains the waxing and waning nature of our patient's mass. Size ranges from a few millimeters to a few centimeters, and the most common location is the lower lip, a likely result of the predilection this area has for trauma by the cuspids.^4^ This lesion is most prevalent in children and young adults in their first 2 decades of life and has a female predominance.^5^

An oral mucocele is classified as either extravasation or retention type, depending on the pathogenesis. Extravasation type is due to mechanical trauma to the excretory duct of the salivary glands, leading to transection or rupture of the duct. Mucus extravasates into the connective tissue stroma, triggering an inflammatory response that leads to the formation of a fibrous pseudocapsule. This type is typically seen in the lower lip, buccal mucosa, and retromolar area. The retention type is due to obstruction of the duct and subsequent buildup of fluid. This type is less common overall and occurs mostly in the elderly.^1^ It can be caused by sialoliths or strictures. Strictures typically result from irritation by tobacco products or mouthwashes.^4^ These lesions typically occur in the upper lip, hard palate, floor of the mouth, or maxillary sinus.

The typical management of oral mucoceles is surgical. Small lesions can be completely excised, along with associated salivary gland tissue, and closed primarily. Larger mucoceles can be treated with marsupialization, since large excisions can carry a risk for dissection of vital structures (ie, labial branch of the mental nerve). Micro-marsupialization is a variation of this technique that is particularly useful for pediatric patients because it is the least traumatic option. It involves incising a cyst and suturing the edges of the subsequent slit along its longest diameter.^6^ Moderate-sized lesions with a thick pseudocapsule can be excised by dissecting the mucocele along with the supplying mucous glands.^4^ Excisions can alternatively be performed with a CO_2_ laser with good outcomes.^3^ Other approaches include cryotherapy and intralesional corticosteroid injection,^7^ but both modalities are associated with a high relapse rate and often require additional interventions.

The likelihood of recurrence after excision is partially dependent on technique and has been reported to occur in 5% to 18% of cases.^3^^,^^6^^,^^8^ This risk can be minimized by removing any peripheral salivary gland projections and placing interrupted marginal sutures to prevent these projections from entering the surgical site.^4^ Furthermore, nearby glands and ducts should be carefully avoided to prevent the occurrence of oral mucoceles secondary to the rupture of adjacent glands. After removal, the specimen should be analyzed histologically to both confirm the diagnosis and ensure that all gland tissue has been removed.

**Figure 1:**
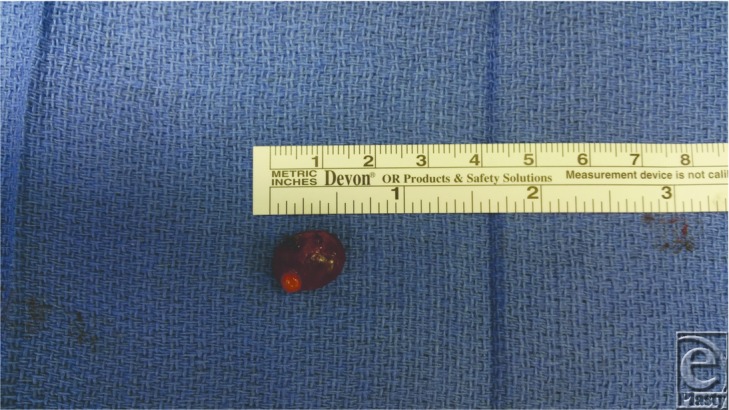
Lower lip mucocele specimen with associated salivary gland tissue.

In our patient, we were able to make an incision directly over the lesion and bluntly dissect the mass out with the psuedocapsule intact along with the associated salivary gland (see Fig 1). After 2-month follow-up, the patient has not had a recurrence.
